# Barasertib: a novel approach for the treatment of metastatic melanoma

**DOI:** 10.1186/1479-5876-12-S1-P7

**Published:** 2014-05-06

**Authors:** Amalia Azzariti, Letizia Porcelli, Anna Elisa Quatrale, Letizia Sidella, RM Iacobazzi, Gabriella Guida, Immacolata Maida, Tiziana Cocco, Anna Ferretta, Sabino Strippoli, Stefania Giuda, Stefani Tommasi, Angelo Paradiso, Michele Guida

**Affiliations:** 1 Clinical Experimental Oncology Laboratory, Istituto Tumori Giovanni Paolo II, Bari, Italy; 2 Dept. of Basic Medical Sciences, Neurosciences and Sense Organs, University of Bari 'A. Moro', 70124 Bari, Italy; 3 Department of Medical Biochemistry, Biology & Physics, University of Bari 'A. Moro', 70124 Bari, Italy; 4 Medical Oncology Department, Istituto Tumori Giovanni Paolo II, Bari, Italy

## Background

Metastatic melanoma represents the most deadly form of skin cancer. The poor response to chemotherapy and the brief response to the anti-BRAF vemurafenib in selected population of patients, make the identification of new therapeutic approaches an urgent need. Our goal is the evaluation of the efficacy of barasertib, an aurora B kinase inhibitor impairing cytokinesis, in both mutated and non-mutated melanoma cell lines.

## Materials and methods

Panel of melanoma cells: BRAFV600E mutated cells (MBA72 and Hmel1), the same cell in which the resistance to vemurafenib was induced by chronic exposure to it (MBA72R and Hmel1R) and BRAF wt (HBL and LND1).

Cells were characterized for vemurafenib and barasertib effectiveness on cell growth by MTT assay after 3 and 6 days of continuous exposure.

Cell cycle was determined by flowcytometry and migration was evaluated by wound-healing assay.

## Results

Cells with BRAFV600E mutation are sensitive to vemurafenib conversely, those with BRAF wt and the resistant ones showed an IC50 of at least 10 folds higher.

3days-barasertib exposure strongly reduced cell growth (30-60% at 30 and 300nM, respectively) in all cell lines; when the drug was given together with vemurafenib, no gain in effectiveness was evident. Prolonged exposure to barasertib (6 days) showed a progressive increase of effectiveness particularly in cells BRAF wt.

The analysis of cell death mechanisms involved in determining the effectiveness of barasertib and vemurafenib showed that the first drug induced both apoptosis and necrosis conversely, the latter mainly apoptosis.

Cell cycle analysis demonstrated that barasertib induced an increase in cell size and in polyploidia, suggesting also the mitotic catastrophe as a further cell death mechanism.

Moreover, the anti-metastatic behaviour of this agent has been evaluated in function of drug concentration and time exposure. Preliminary results showed a strong reduction of cell migration after drug exposure.

## Conclusions

The sensitivity of melanoma cells to barasertib is irrespective to BRAF mutational status; however, cells BRAF wt show an higher nuclear modification. In conclusion, our results suggest barasertib as a novel therapeutic approach in melanoma treatment irrespective of BRAFV600E mutation.

